# Genomic Characterization of Two *Escherichia fergusonii* Isolates Harboring *mcr-1* Gene From Farm Environment

**DOI:** 10.3389/fcimb.2022.774494

**Published:** 2022-05-26

**Authors:** Ruishan Liu, Hao Xu, Xiaobing Guo, Shuxiu Liu, Jie Qiao, Haoyu Ge, Beiwen Zheng, Jianjun Gou

**Affiliations:** ^1^ Department of Laboratory Medicine, The First Affiliated Hospital of Zhengzhou University, Zhengzhou, China; ^2^ State Key Laboratory for Diagnosis and Treatment of Infectious Diseases, The First Affiliated Hospital, College of Medicine, Zhejiang University, Hangzhou, China; ^3^ Department of Laboratory Medicine, The Fifth Affiliated Hospital of Zhengzhou University, Zhengzhou, China

**Keywords:** IncX4, IncI2, farm environments, whole-genome sequencing, core genome analysis

## Abstract

The prevalence and transmission of mobile colistin resistance (*mcr*) genes have led to a severe threat to humans and animals. *Escherichia fergusonii* is an emerging pathogen which is closely related to a variety of diseases. However, the report of *mcr* genes harboring *E. fergusonii* is still rare. One study in Brazil reported the *E. fergusonii* isolates with IncHI2-type plasmids harboring *mcr-1*. A Chinese study reported two strains carrying *mcr-1* gene with the same plasmid type IncI2. Here, we identified two strains of *E. fergusonii* carrying *mcr-1* gene from farm environments with IncX4-type and IncI2-type plasmids, respectively. To our best knowledge, this is the first report about *mcr-1* gene located on IncX4-type plasmid in *E. fergusonii*. We investigate the resistance mechanism of colistin-resistant *Escherichia fergusonii* strains 6S41-1 and 5ZF15-2-1 and elucidate the genetic context of plasmids carrying *mcr-1* genes. In addition, we also investigated chromosomal mutations mediated colistin resistance in these two strains. Species identification was performed using MALDI-TOF MS and 16S rRNA gene sequencing. The detection of *mcr-1* gene was determined by PCR and Sanger sequencing. S1-pulsed-field gel electrophoresis (PFGE), Southern blotting, antimicrobial susceptibility testing, conjugation experiments, complete genome sequencing, and core genome analysis were conducted to investigate the characteristics of isolates harboring *mcr-1*. The *mcr-1* genes on two strains were both plasmids encoded and the typical IS*26*-*parA*-*mcr-1*-*pap2* cassette was identified in p6S41-1 while a *nikA*-*nikB*-*mcr-1* locus sites on the conjugative plasmid p5ZF15-2-1. In addition, Core genome analysis reveals that *E. fergusonii* 6S41-1 and 5ZF15-2-1 have close genetic relationships. The *mcr-1* gene is located on conjugative IncI2-type plasmid p5ZF15-2-1, which provides support for its further transmission. In addition, there’s the possibility of *mcr-1* spreading to humans through farm environments and thereby threatening public health. Therefore, continuous monitoring and investigations of *mcr-1* among *Enterobacteriaceae* in farm environments are necessary to control the spread.

## Introduction

*Escherichia fergusonii* is a species with high genotypic and phenotypic similarity to *Escherichia coli*. DNA hybridization shows that it has 64% similarity with *Escherichia coli-Shigella* (Farmer et al., 1985a). It was once known as Enteric Group 10 until Farmer et al. proposed it as a new species within the genus *Escherichia* and family *Enterobacteriaceae* in 1985 (Farmer et al., 1985b). Since then, *E. fergusonii* has been considered as a significant emerging opportunistic pathogen both for animals and humans. *E. fergusonii* was initially isolated from blood samples of clinical patients (Farmer et al., 1985b). To this day, *E. fergusonii* has been widely isolated from animals, humans, and the environment. In specimens of animal origin, it has been isolated from the feces of goats, sheep, horses, cattle, pigs, chickens, turkeys, reindeer, and ostriches that display symptoms of salmon-like infection including diarrhea, meningitis, mastitis, abortion, and septicemia (Farmer et al., 1985b; Bain and Green, 1999; Herráez et al., 2005; Hariharan et al., 2007; Foster et al., 2010; Oh et al., 2012). While in clinical specimens, *E. fergusonii* is often isolated from blood, urine, feces, gallbladder fluid, spinal fluid, and abdominal wound samples of patients with septicemia, intestinal diseases, urinary tract infections, and pancreatic cancer (Farmer et al., 1985b; Funke et al., 1993). In addition, *E. fergusonii* was also isolated from food products during routine screening procedures and water samples (Fegan et al., 2006; Maifreni et al., 2013; Maheux et al., 2014).

Colistin is a group of polypeptide antibiotics produced by *Paenibacillus polymyxa*, which was first discovered in the 1940s and is considered to be the last line of defense against severe infection caused by pan-drug-resistant Gram-negative pathogens (Storm et al., 1977; Sun et al., 2018). For decades, colistin has been used in curative treatments and is widely regarded as a preventive drug in veterinary medicine (Skov and Monnet, 2016). However, the highly selective pressure caused by the extensive use of colistin in animals has led to the selection and the spread of colistin-resistant pathogens (Asai et al., 2005; Diarra et al., 2007). The resistance mechanism of *Enterobacteriaceae* to colistin could be mediated both by chromosomes and plasmids (Huang et al., 2020). For a long period, the resistance was thought to be caused by chromosomal mutations such as two-component systems and its regulators, or the overexpression of efflux pump proteins. None of these were transferable (Anyanwu et al., 2020). However, since the first discovery of mobile colistin resistance gene *mcr-1* in *E. coli* from China (Liu et al., 2016), 10 different *mcr* genes and several variants have been identified across the globe which demonstrates a horizontal transfer mechanism for colistin resistance (Luo et al., 2017; Wang et al., 2020a). The emergence of *mcr* genes has aroused widespread attention and concern around the world. Although China banned colistin as a feed additive for animals in 2017 (Walsh and Wu, 2016), the problem of antibiotic resistance caused by *mcr* genes is still severe (Wang et al., 2020b).

Among the 10 different *mcr* genes, *mcr-1* is most widespread and commonly detected in *Enterobacteriaceae*, especially *Escherichia coli* (Jeannot et al., 2017). However, the detection of *mcr-1* gene in *E. fergusonii* is still rarely reported, which is worthy of our attention and further study. In addition, the multi-drug resistance and even pan-drug resistance pathogens caused by the coexistence of *mcr-1* and other drug resistance genes make the option of anti-infective therapy in a dilemma and pose a serious threat to public health. Here, we identified two strains of *E. fergusonii* carrying *mcr-1*, which were isolated from farm soils and feces of a healthy pig, respectively, and performed core genome analysis by whole-genome sequencing data. Furthermore, we studied the drug resistance profile, plasmid characteristics, and chromosomal mutations to reveal the potential resistance mechanism of these two strains.

## Material And Methods

### Strain Screening

In a routine surveillance study of antimicrobial resistance of bacteria from farm environments, we collected samples from conventional farms since May 2019. All samples were collected using sterile cotton swabs and were kept in -20°C during transportation. And all samples were enriched within 72 h after sampling. The enriched samples were coated on MacConkey agar (OXOID, Hampshire, United Kingdom) plates containing 2 mg/L colistin for preliminary screening. Identification of species was performed using both matrix-assisted laser desorption/ionization time-of-flight mass spectrometry (MALDI-TOF/MS) (Bruker Daltonik GmbH, Bremen, Germany) and 16S rRNA gene sequencing. The *mcr-1* gene was detected under PCR and Sanger sequencing, as described previously (Zheng et al., 2015).

### Location of *mcr-1* Gene and Transferability of Plasmids Carrying *mcr-1*

The size and number of plasmids of *E. fergusonii* 6S41-1 and 5ZF15-2-1 were identified by the S1-PFGE. In addition, the location of *mcr-1* gene was determined according to Southern blotting and hybridization with digoxigenin-labeled *mcr-1* specific probe. The transferability of the plasmid carrying *mcr-1* gene was verified by conjugation experiments using rifampicin-resistant *E. coli* 600 as the recipient strain according to the previous study (Zheng et al., 2017). The transconjugants were selected on Mueller-Hinton medium containing 200mg/L rifampicin and 1mg/L colistin. Then, the transconjugants were identified by MALDI-TOF/MS, and the *mcr-1* gene was detected by PCR to confirm whether the plasmids were successfully transferred into the recipients.

### Antimicrobial Susceptibility Testing

The antimicrobial susceptibility profiles of 6S41-1 and 5ZF15-2-1, and their corresponding transconjugants, were determined using the agar dilution method and broth microdilution method. The results were interpreted according to Clinical and Laboratory Standards Institute (CLSI) standards (https://clsi.org), except colistin and tigecycline which are interpreted according to EUCAST clinical breakpoints (https://www.eucast.org/). *E. coli* ATCC 25922 was used as a quality control.

### Whole Genome Sequencing and *In Silico* Analyses

Genomic DNA was extracted by using a Bacterial DNA Kit (QIAGEN, Hilden, Germany). The harvested DNA was detected by the agarose gel electrophoresis and quantified by Qubit^®^ 2.0 Fluorometer (Thermo Scientific). Then the DNA was sequenced both on the Illumina NovaSeq 6000 (Illumina, San Diego, CA, United States) and Oxford Nanopore platforms (Oxford Nanopore Technologies, Oxford, United Kingdom) to obtain short-read data and long-read data, respectively. The raw llumina reads were assembled using SPAdes3.10.0, and then the sequencing results were hybrid assembled with Unicycler v0.4.7 to get the complete genome sequence (Wick et al., 2017). The bacterial genomes were annotated using Prokka. Additionally, the acquired antimicrobial resistance genes and replicon type of plasmid were determined using online tools (http://www.genomicepidemiology.org/), while the transposon and IS elements were identified using the ISFinder database (http://www-is.biotoul.fr/). The virulence genes of isolates were identified using VFDB. Finally, the circular image of multiple plasmids comparisons was plotted by the BLAST Ring Image Generator (BRIG) (Alikhan et al., 2011). The comparison figure of the genetic environment surrounding the *mcr-1* gene was generated by Easyfig 2.2.3 (Sullivan et al., 2011).

### Chromosomal Mutations Mediated Colistin Resistance

Based on WGS data, amino acid sequences of isolates 6S41-1 and 5ZF15-2-1 were compared with reference strain *E. fergusonii* RHB19-C05. PROVEAN (http://provean.jcvi.org/index.php) was used to predict whether the amino acid substitutions in two-component systems, AcrAB-TolC pump system and its regulators, affect protein function. Additionally, SMART analysis (http://smart.embl-heidelberg.de/) was performed to determine the corresponding domain architectures.

### Core Genome Analysis

Genome sequences for 114 strains of *E. fergusonii* were downloaded from the NCBI database, assembly section (**Table S4**). According to Wu *et al.* (Wu et al., 2021), the inclusion of confounding strains may introduce important biases. Thus, we performed average nucleotide identity (ANI) analysis using pyani (https://github.com/widdowquinn/pyani). Then these genomes, plus *E. fergusonii* 6S41-1 and 5ZF15-2-1, were performed phylogenetic analyses using Roary (Page et al., 2015), a tool that builds rapid large-scale prokaryote pan genomes and identifies core genes. Next, a maximum likelihood phylogenetic tree was generated by MEGA X using core genes. And the visualization and modification were performed by iTOL (https://itol.embl.de/).

## Results

### Isolation and Identification of *E. fergusonii* 6S41-1 and 5ZF15-2-1 Harboring *mcr-1*

In total, we collected 80 soil samples and 534 fecal samples from six randomly selected farms in Jiaxing, Zhejiang Province. Among them, two distinct colistin-resistant *E. fergusonii* isolates were identified and named 6S41-1 and 5ZF15-2-1, respectively. In more detail, strains 6S41-1 and 5ZF15-2-1were isolated from the soils and the feces of a healthy pig in two different farms, respectively. The single colonies selected from the selective mediums were identified as *E. fergusonii* using MALDI-TOF/MS and 16S rRNA, and the *mcr-1* gene was confirmed by PCR and sequencing.

### Antimicrobial Susceptibility Profiles

A total of 17 antibiotics were included in this study (**Table 1**). The AST revealed that isolate 6S41-1 exhibited resistance to colistin, gentamicin, and chloramphenicol but was shown to be susceptible to tigecycline, amoxicillin-clavulanate, piperacillin/tazobactam, ceftazidime, ceftriaxone, cefepime, cefotaxime, imipenem, meropenem, trimethoprim/sulfamethoxazole, amikacin, and aztreonam. In addition, the minimum inhibitory concentration (MIC) values of ciprofloxacin and levofloxacin for 6S41-1 were determined as intermediate. Strain 5ZF15-2-1 was sensitive to almost all antibiotics except colistin, with MIC value of 4 mg/L. Moreover, the transconjugants 5ZF15-2-1-*E. coli* 600 showed the same antibiotic resistance profile to 5ZF15-2-1 but was intermediate to colistin.

**Table 1:** MIC values of antimicrobials for *E. fergusonii* 6S41-1, 5ZF15-2-1, transconjugant 5ZF15-2-1-*E. coli* 600 and recipient strain *E. coli* 600.

Antimicrobials	MIC values (mg/L)			
	*E. fergusonii *6S41-1	*E. fergusonii *5ZF15-2-1	5ZF15-2-1-*E. coli* 600	*E. coli* 600
Amoxicillin/clavulanate	4/2	4/2	8/4	8/4
Piperacillin/tazobactam^a^	1/4	0.25/4	2/4	4/4
Ceftazidime	0.25	0.25	0.5	0.5
Ceftriaxone	≤0.03	≤0.03	≤0.03	≤0.03
Cefepime	≤0.008	≤0.008	≤0.008	≤0.008
Cefotaxime	0.03	0.03	0.06	0.03
Ciprofloxacin	0.5	≤0.004	0.25	0.25
Levofloxacin	1	0.03	0.25	0.25
Imipenem	0.125	0.125	0.5	0.5
Meropenem	0.015	0.015	0.03	0.03
Trimethoprim/ sulfamethoxazole	0.125/2.375	0.125/2.375	0.125/2.375	0.125/2.375
Amikacin	>4	4	2	2
Gentamicin	128	1	0.5	0.5
Aztreonam	0.06	≤0.03	0.06	0.125
Chloramphenicol	>64	4	4	4
Colistin	8	4	2	1
Tigecycline	0.125	≤0.03	≤0.03	≤0.03

a Tazobactam at a fixed concentration of 4mg/L.

### Genomic and Virulence-Associated Features of *E. fergusonii* 6S41-1 and 5ZF-15-2-1

The genomic features of *E. fergusonii* 6S41-1 and 5ZF15-2-1 are displayed in **Table 2**. *E. fergusonii* 6S41-1 genome consists of a 4,724,978 bp circular chromosome with an average G+C content of 49.8% and six plasmids. The size and average G+C content of the plasmid conferring *mcr-1* was 46,439 bp and 44.4%, respectively. The genome of 5ZF15-2-1 consists of a chromosome of 4,934,492 bp and four plasmids The plasmid types of these two strains that do not carry *mcr-1* gene are shown in **Table S1**. A screening for acquired resistance determinants found that no resistance gene was encoded on the chromosome, and only one resistance gene, *mcr-1*, was encoded on the plasmid p5ZF15-2-1. Moreover, plasmid p6S41-1 encoding acquired resistance genes both *mcr-1* and *qnrS1*, the latter mediating resistance to ciprofloxacin (**Table S2**). Virulome analysis showed that the majority of virulence genes are encoded on chromosomes in both strains only *cseA* which encoded adhesin protein sited on a IncFII type plasmid of *E. fergusonii* 6S41-1 (**Table S1**). In particular, the virulence profiles of these two strains were almost identical. For instance, they both contained virulence factors encoded secretions systems (*tssALMJ*, *clpV/tssH* and *hcp2/tssD2*), transcriptional regulators (*rcsB*), and outer membrane proteins (*ompA*).

**Table 2:** Genomic features of the *E. fergusonii* 6S41-1 and 5ZF15-2-1.

Feature	*E. fergusonii* 6S41-1		*E. fergusonii* 5ZF15-2-1	
	chromosome	p6S41-1	chromosome	p5ZF15-2-1
Size (bp)	4,724,978	46,394	4,934,492	61,228
G + C content (%)	49.8	44.4	49.8	42.4
No. of protein-coding sequences	4,322	135	4,573	75
No. of tRNA genes	87	0	91	0
No. of rRNA genes	22	0	22	0
Plasmid replicon type	–	IncX4	–	IncI2
Resistance genes	–	*mcr-1.1*, *qnrS1*	–	*mcr-1.1*
Accession numbers	CP079884	CP079887	CP079891	CP079893

### Characterization of Plasmids Bearing *mcr-1*

**Figure 1:**
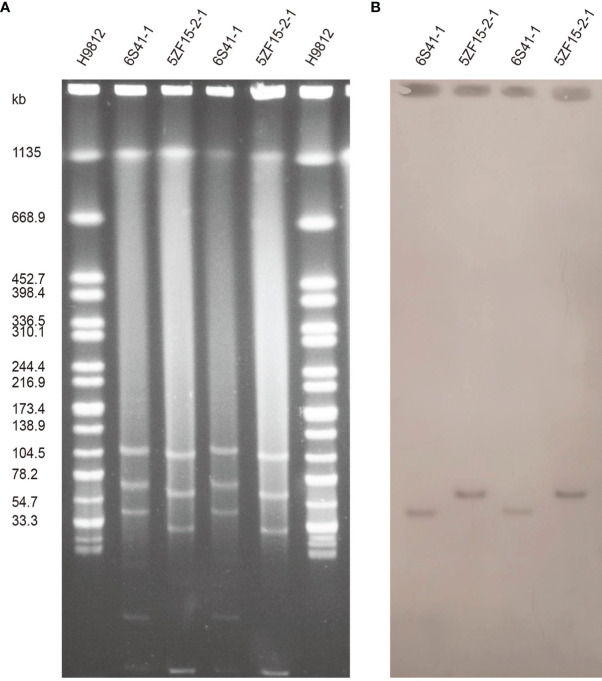
Plasmid profiles of *E. fergusonii* 6S41-1 and 5ZF15-2-1. **(A)** Plasmid size determination by S1-PFGE, with *Salmonella enterica serotype* Braenderup H9812 as the size marker. **(B)** Southern blotting hybridization with an *mcr-1*-specific probe.

**Figure 2:**
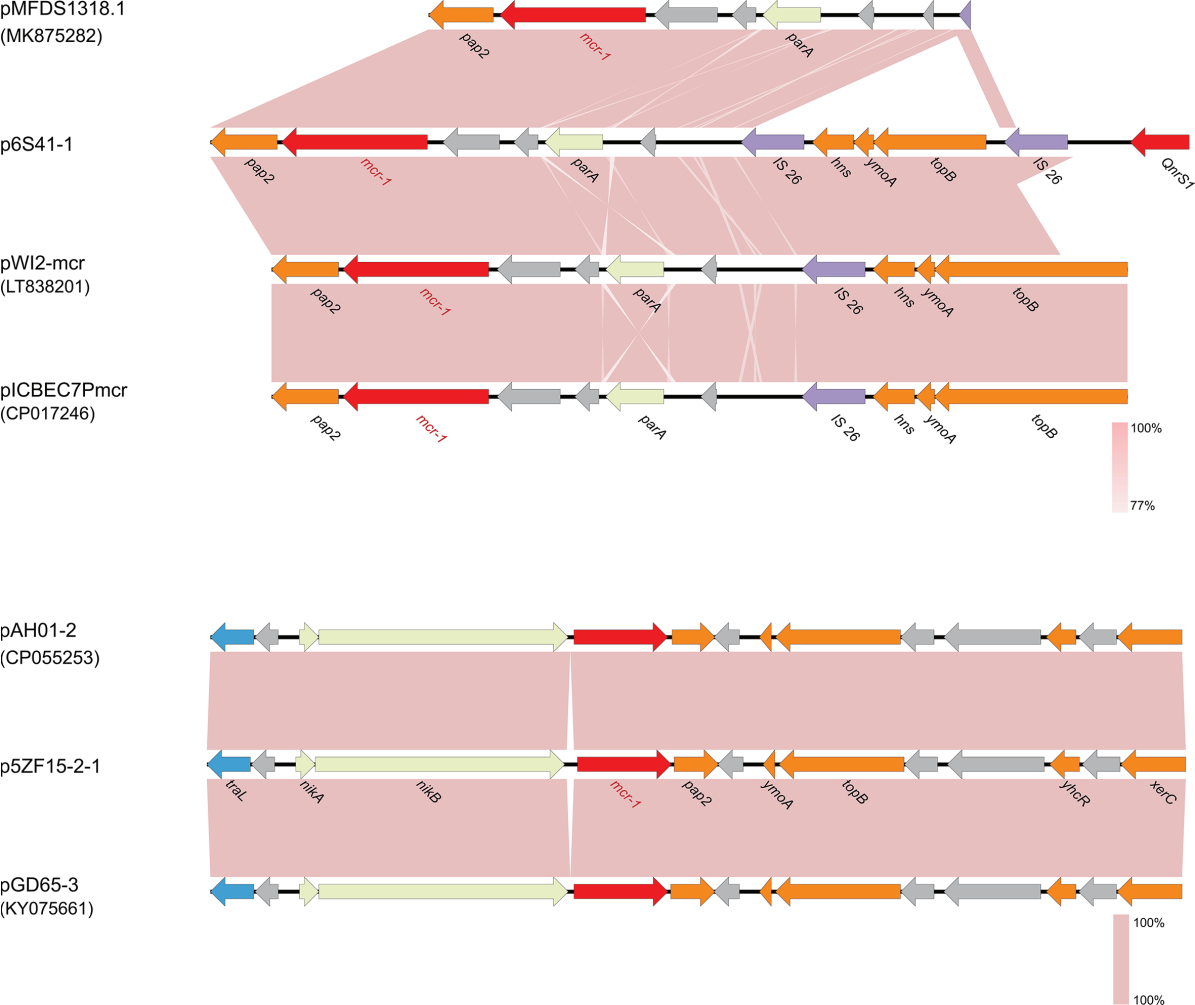
The genetic context of *mcr-1* gene on p6S41-1 and p5ZF15-2-1. **(A)** Comparison of genes surrounding *mcr-1* on p6S41-1, pMFDS1318.1 (accession number: MK875282), pWI2-mcr (accession number: LT838201) and pICBEC7Pmcr (accession number: CP017246). **(B)** Comparison of genes surrounding *mcr-1* on p5ZF15-2-1, pAH01-2 (accession number: CP055253) and pGD65-3 (accession number: KY075661). Open reading frames (ORFs) are shown as arrows and indicated according to their putative functions. Purple indicates genes related to mobile elements, red indicates genes related to drug resistance, light yellow indicates genes related to plasmid stability, blue indicates genes involved in conjugation and orange represents other functional genes. Hypothetical protein encoded genes are colored by grey. Regions with a high degree of homology are indicated by pink shading.

S1-PFGE and Southern blot confirmed that 6S41-1 isolate contained a ~46 kb plasmid harboring *mcr-1* gene (**Figure 1**). The WGS results demonstrated that the *mcr-1*-encoding plasmid, designated as p6S41-1, was an IncX4-type plasmid with the size of 46,394 bp and contained 135 protein-coding genes with a GC content of 44.4% (**Table 2**). We tried to transfer plasmid p6S41-1 to the recipient strain *E. coli 600*. However, *in vitro* conjugation experiments were unsuccessful. We also tried to transfer plasmid extracted from isolate, and repeated transformation methods failed to move the plasmid to recipient *E. coli* DH5α cells. p6S41-1 contains a collection of genes involved in segregation, stability, replication, and conjugative transfer of the plasmid (*dnaJ*, *pir*, *parA*, *hns*, *topB* and *virB4,8,9,11*), which together constructed the basic backbone of the plasmid. The genetic context of *mcr-1* was characterized by an IS*26* element upstream of the *mcr-1-pap2* element, and similar structures could be seen in *E. coli* plasmid pMFDS1318.1 (accession number: MK875282), *E. coli* plasmid pWI2-mcr (accession number: LT838201), and *E. coli* plasmid pICBEC7Pmcr (accession number: CP017246) (**Figure 2A**).

The plasmid harboring *mcr-1* of *E. fergusonii* 5ZF15-2-1 was 61,228 bp in length with 75 CDSs and a GC content of 42.4% (**Table 2**). *In silico* analysis indicated that plasmid p5ZF15-2-1 belongs to IncI2. Conjugation experiments revealed that the transmission of *mcr-1* from *E. fergusonii* 5ZF15-2-1 to *E. Coli* 600 was successful, which was consistent with the antimicrobial susceptibility profile of the transconjugant (**Table 1**). Annotation revealed that *mcr-1* was the only resistance gene on plasmid p5ZF15-2-1, which carries genes coding for replication initiation and conjugative transfer assembly proteins, plasmid stability proteins, and other functional proteins. Exploration of the genetic context surrounding *mcr-1* showed that the genes *traL*, *nikA*, and *nikB* were present in the upstream region, and the genes *pap2*, *ymoA*, *topB*, *yhcR*, and *xerC* were present in the downstream region. As shown in **Figure 2B**, the sequence encompassing the *traL*-*hp*-*nikA*-*nikB*-*mcr*-*1*-*pap2*-*hp*-*ymoA*-*topB*-*hp*-*hp*-*yhcR*-*hp*-*xerC* region in this study showed a 100% nucleotide identity with the corresponding region of the IncI2 plasmids pAH01-2 (accession number: CP055253) and pGD65-3 (accession number: KY075661) which both *E. coli* strains and isolated from Anhui and Guangzhou, respectively. Further, plasmid p5ZF15-2-1 carried genes encoding pilus and conjugative transfer proteins *pilU*, *pilT*, *pilQ*, *pilO*, which provided evidence for transmission of the plasmid.

**Figure 3:**
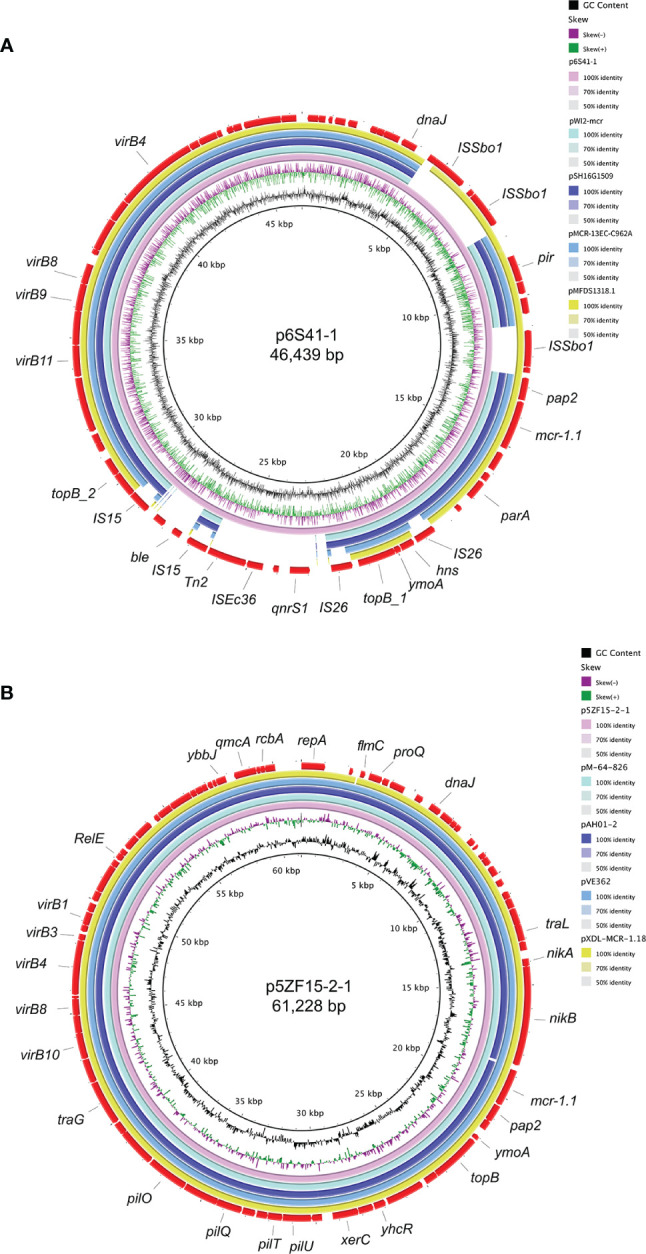
The genetic features of plasmid p6S41-1 and p5ZF15-2-1. **(A)** Circular comparison between *mcr-1* bearing IncX4 plasmids p6S41-1 in this study and four similar IncX4 plasmids in NCBI nr/nt database. p6S41-1 was used as the reference in the outermost ring. **(B)** Circular comparison between *mcr-1* bearing IncI2 plasmids p5ZF15-2-1 in this study and four similar IncI2 plasmids in NCBI nr/nt database. p5ZF15-2-1 was used as the reference in the outermost ring.

By the search of the nr/nt database, plasmid p6S41-1 in our study was closely related to *E. coli* pWI2-mcr (accession number: LT838201), *S. enterica* pSH16G1509 (accession number: MK477615), *E. coli* pMCR-13EC-C962A (accession number: KX555452), and *E. coli* pMFDS1318.1 (accession number: MK875282), from the same incompatibility. Further, plasmid p5ZF15-2-1 exhibiting 99% nucleotide identity and 99%-100% query coverages to pM-64-826 (accession number: MT773675), pAH01-2 (accession number: CP055253), pVE362 (accession number: AP018355), and pXDL-MCR-1.18 (accession number: CP043036). As shown in **Figure 3**, the backbone sequences of these plasmids were almost identical.

### Amino Acid Variants on Chromosome

The results of amino acid sequences comparison show that amino acid mutations occurred in PmrA, PhoQ, and MgrB (**Table S3**). Among them, the substitution T299I in PhoQ was predicted to affect protein functions according to PROVEAN. In more detail, it might have affected phosphate transfer for it is located on His Kinase A (phosphoacceptor) domain which is the key element in two-component signal transduction systems according to SMART analysis. In accordance with the results of AST, the MIC of colistin for transconjugants 5ZF15-2-1-*E. coli* 600 was lower than that of 5ZF15-2-1 which indicated that isolate 5ZF15-2-1 may acquire colistin resistance *via* chromosomal mutation on PhoQ besides *mcr-1* gene.

### Core Genome Analysis

**Figure 4:**
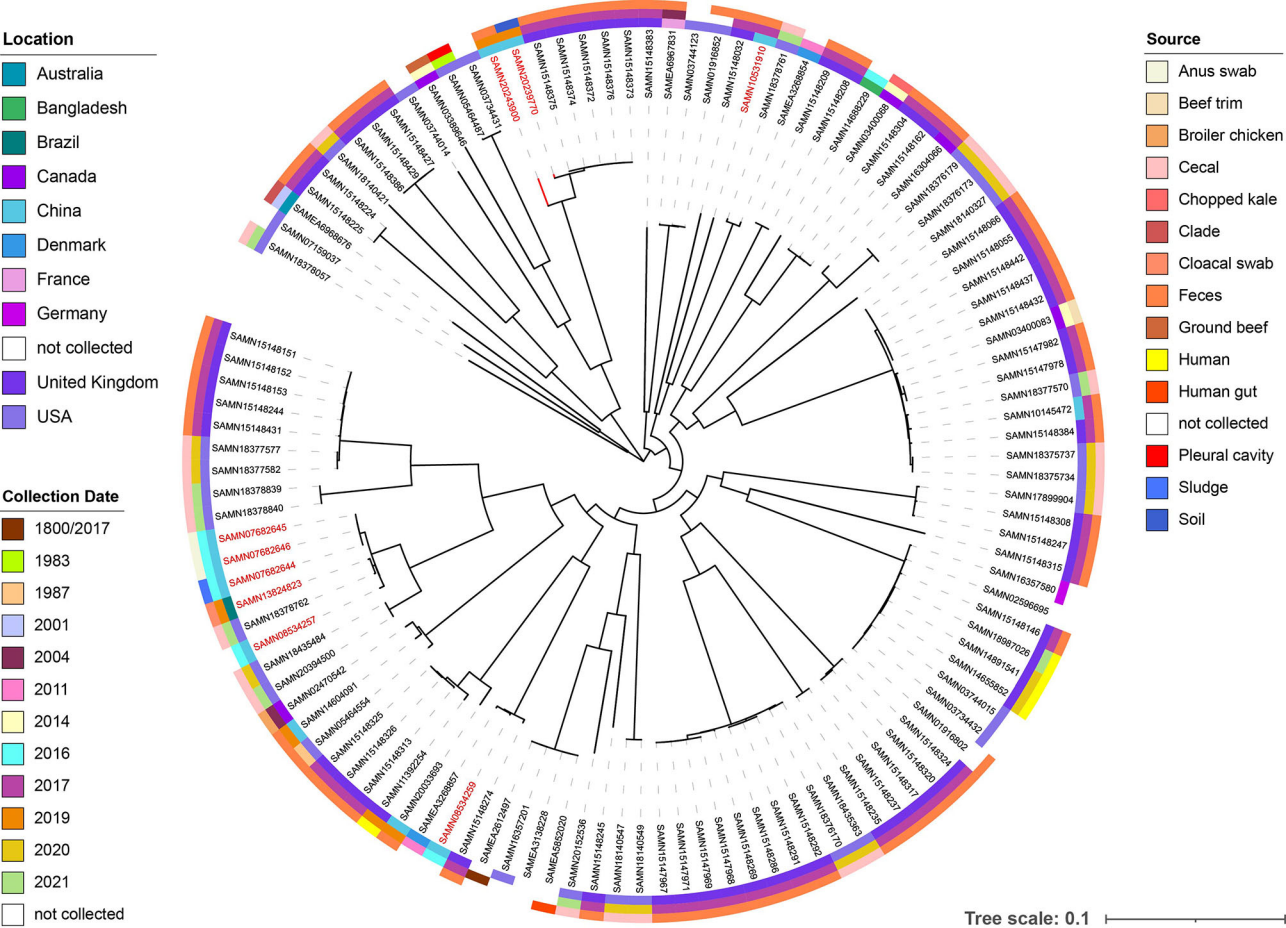
The maximum likelihood phylogenetic tree based on the core genome sequences of 115 *E. fergusonii* strains. *E. fergusonii* 6S41-1 (BioSample: SAMN20239770) and 5ZF15-2-1 (BioSample: SAMN20243900) are indicated in red. The other *mcr-1*-bearing strains were marked by dark red. The three circles around the phylogenetic tree indicate the location (inner circle), collection date and source (outer circle) of these strains.

According to the result of ANI analysis, one strain (SAMEA5771506, **Figure S1**) was removed in subsequential analyses. The result of phylogenetic analysis of 115 core genomes (**Figure 4**) shows strains 6S41-1 (BioSample: SAMN20239770) and 5ZF15-2-1 (BioSample: SAMN20243900) in this study exhibited close phylogenetic relationships. In addition, the two strains are far from the other nine strains from China (SAMN20033693, SAMN14604091, SAMN10145472, SAMN08534259, SAMN07682644, SAMN07682645, SAMN07682646, SAMN08534257, SAMN10531910) but closely related to (SAMN15148372, SAMN15148373, SAMN15148374, SAMN15148375, SAMN15148376) from the United Kingdom. The five strains from the United Kingdom were all isolated from sheep feces on a farm in 2017. Further, we conducted drug resistance gene analysis on 113 strains from the database *via* Resfinder and found that seven strains carried *mcr-1*. Interestingly, six of seven strains were isolated from China and came from a variety of sources including sludge, anus swab, and feces, which indicates China may be a main reservoir of *mcr-1*-bearing *E. fergusonii* (**Figure 4**). In terms of the phylogenetic tree, the nine strains (including two in this study) from China could be divided into four clusters and are distantly related.

## Discussion

*Enterobacteriaceae* is one of the leading causes of both nosocomial and community-acquired infections. And genus *Escherichia* is the most important one among it. *E. fergusonii*, as the emerging pathogen of genus *Escherichia* in recent years, is increasing in prevalence and spread among humans and animals worldwide (Glover et al., 2017; Adesina et al., 2019). It was reported that infections caused by *E. fergusonii* have occurred in many countries (Savini et al., 2008; Lai et al., 2011). In addition, according to recent reports, the resistance of *E. fergusonii* to antibiotics is increasingly observed. For example, *E. fergusonii* has been found harboring β-lactams genes and carbapenemase-resistant genes, which contribute significantly to the growing clinical drug selection burden (Savini et al., 2008; Lagacé-Wiens et al., 2010). However, only a few studies have reported *mcr-1* gene in *E. fergusonii* (da Silva Pontes et al., 2020), and the phylogenetic analysis of *E. fergusonii* is still lacking. Therefore, we conducted core genome analysis of *E. fergusonii* 6S41-1 and 5ZF15-2-1 by whole-genome sequencing data. In addition, we also elucidated the molecular characteristics and genetic context of plasmids carrying *mcr-1* gene.

Colistin was reconsidered as a valid therapeutic option with the increasing carbapenem-resistant bacteria (Bialvaei and Samadi Kafil, 2015). But the emergence of mobile colistin resistance gene *mcr-1* threatens public health and aroused concern over the world. To date, *mcr-1* has been found in more than 50 countries and regions across six continents and has been observed in 15 bacterial species, including *Acinetobacter baumannii*, *Enterobacter cloacae*, *E. coli*, *Klebsiella aerogenes*, and so on (Xiaomin et al., 2020). In this study, we identified two strains of *E. fergusonii* carrying *mcr-1* and performed core genome analysis. The results showed that *E. fergusonii* 6S41-1 and 5ZF15-2-1 have close genetic relationships with strains (SAMN15148375, SAMN15148373, SAMN15148372, SAMN15148376, SAMN15148376) for they all belong to the same major cluster and all isolated from farm environment. On the other hand, *E. fergusonii* has been isolated from different sources and has been detected in different countries and at different times. The main source and country are feces and the United Kingdom, respectively.

In this study, the typical IS*26*-*parA*-*mcr-1*-*pap2* cassette was identified in plasmid p6S41-1, which is similar to other IncX4 plasmids (**Figure 2A**) (Du et al., 2020). In addition, p6S41-1 did not have an IS*Apl1* insertion sequence upstream of the *mcr-1* gene, which is in accordance with other studies (Sun et al., 2017). Generally, IS*Apl1* played a pivotal role in the mobilization of *mcr-1* (Snesrud et al., 2017). However, IS*Apl1* in front of *mcr-1* was absent in all IncX4-type plasmids (Sun et al., 2017). Reports provided evidence that IS*Apl1* is involved in the initial transposition of the *mcr-1* element and then is lost for the stability of *mcr-1* on IncX4 plasmids (Li et al., 2017; Snesrud et al., 2017). In similarity to some other known IncI2-type plasmids (Wang et al., 2017), IS*Apl1* was also absent on plasmid p5ZF15-2-1. And a *nikA*-*nikB*-*mcr-1* locus sites on p5ZF15-2-1. The same structure was also present in studies by Zheng et al. and Wang et al. (Wang et al., 2017; Zheng et al., 2019). Wang et al. speculated these IncI2 plasmids have contributed to the spread of *mcr-1* genes among different species *via* horizontal means by diversified conjugation-aided mechanisms without the assistance of the IS*Apl1* gene (Wang et al., 2017). This hypothesis was consistent with the results of conjugation assay in this study. Interestingly, the chromosomal mutations T299I in PhoQ of *E. fergusonii* 6S41-1 was predicted to affect protein function which might contribute to colistin resistance besides *mcr-1* gene. This suggests that multiple drug resistance mechanisms co-exist in *E. fergusonii* 6S41-1. In addition, the *pap2* gene, which can be found in both these two plasmids, was confirmed as necessary along with *mcr-1* to reduce colistin susceptibility (Choi et al., 2020).

Different replicons have been found carrying *mcr-1* since the first report in 2016 (Liu et al., 2016), including IncI2, IncX3, IncX4, IncF, IncFII, IncH1, IncHI1, IncHI2, IncP, and IncY (Hu et al., 2021). Most plasmids carrying *mcr-1* proved to be transferable, with IncX4 and IncI2 as dominant types among them (Xiaomin et al., 2020). To date, IncX4 and IncI2 plasmids carrying *mcr-1* have been identified in different species of *Enterobacteriaceae*, including *K. pneumoniae*, *Salmonella*, *E. coli*, *C. braakii*, and *C. sakazakii*, which come from various sources, including humans, food animals, and animal production environments (Zhou et al., 2017; Wang et al., 2018; Zheng et al., 2019; Xiaomin et al., 2020). A previous study reported the *E. fergusonii* isolates with IncHI2-type plasmids harboring *mcr-1* in Brazil (da Silva Pontes et al., 2020). That same year, a Chinese study reported two strains carrying *mcr-1* gene isolated from caecal contents of chickens with the same plasmid type IncI2 (Li et al., 2020). In this study, we isolated two strains of *E. fergusonii* carrying *mcr-1* which the plasmids types were IncX4 and IncI2, respectively. To our best knowledge, this is the first report about *mcr-1* gene located on IncX4-type plasmid in *E. fergusonii*. It demonstrated the further spread of *mcr-1* with different replicons among different species of *Enterobacteriaceae*, indicating the need to pay more attention to it. Of note, these two plasmids, both carrying genes encoding type IV secretion systems, could increase bacterial competitiveness and may contribute to the fitness of the antibiotic-resistant bacteria.

According to the “One Health” concept, the environment plays a vital role in safeguarding public health. And environmental AMR was identified as six emerging issues of greatest concern by the United Nations Environment Programme (UNEP) (Anyanwu et al., 2020). According to current reports, *mcr-1* bearing isolates have been detected in various farm environments, including *E. coli* strains isolated from dairy cow feces in China with *mcr-1* genes encoded on IncI2-type plasmid and chromosomes respectively, *mcr-1* bearing *E. coli* strains isolated from farm soils in China in 2017 with various of plasmids types and MLST types, and *mcr-1*-positive strains from manure, swabs, and flies in German swine farms (Guenther et al., 2017; Zheng et al., 2017; Zheng et al., 2019). This indicates that farm environments are important reservoirs for the storage and spread of *mcr-1*. This result is most likely due to the fact that colistin is used as a feed additive to promote animal growth. In this study, *E. fergusonii* isolates 6S41-1 and 5ZF15-2-1 carrying *mcr-1* from farm soils and pig feces imply the extended dissemination of *mcr-1* among different species of *Enterobacteriaceae* in farm environments, indicating the urgent need for surveillance and further study of *mcr-1* gene in farm environments.

## Conclusion

In summary, we identified two *E. fergusonii* isolates harboring *mcr-1* and described the complete sequence by whole-genome sequencing. The *mcr-1* genes located on IncX4 and IncI2 plasmids spread widely among species of *Enterobacteriaceae* worldwide. Not only that, the *mcr-1* gene located on IncI2-type plasmid p5ZF15-2-1 has the ability of horizontal transfer, which provides support for its further transmission. For *mcr-1* gene can spread *via* food chains, the source of two strains highlights the possibility that *mcr-1* may spread to humans through farm environments and thereby threaten public health. Therefore, continuous monitoring and investigations of *mcr-1* in farm environments are necessary to control its spread.

## Data Availability Statement

The datasets presented in this study can be found in online repositories: https://www.ncbi.nlm.nih.gov/ (accession numbers CP079884-CP079890).

## Author Contributions

JG and BZ conceived and designed the experiments. RL, SL, JQ, and HG collected samples and performed the experiments. HX and XG analyzed the data. RL wrote the manuscript. All authors contributed to the article and approved the submitted version.

## Funding

This work was supported by research grants from Henan Science and Technology Department (No. 192102310059), the National Key Research and Development Program of China (No.2016YFD0501105), the National Natural Science Foundation of China (No. 82072314), and Henan Province Medical Science and Technology Research Project Joint Construction Project (No. LHGJ20190232).

## Conflict of Interest

The authors declare that the research was conducted in the absence of any commercial or financial relationships that could be construed as a potential conflict of interest.

## Publisher’s Note

All claims expressed in this article are solely those of the authors and do not necessarily represent those of their affiliated organizations, or those of the publisher, the editors and the reviewers. Any product that may be evaluated in this article, or claim that may be made by its manufacturer, is not guaranteed or endorsed by the publisher.
