# Supplementary material for: Genomic Characterization of Two Escherichia fergusonii Isolates Harboring mcr-1 Gene From Farm Environment
Source: Front Cell Infect Microbiol. 2022 May 26;12:774494. doi: 10.3389/fcimb.2022.774494 (PMC9204285; doi:10.3389/fcimb.2022.774494)

Supplementary Material

**Table S1** The virulence-associated features and non-*mcr-1*-bearing plasmids types of two strains.

|  | Virulence Factors | Site | Hit description | Plasmid replicon type |
| --- | --- | --- | --- | --- |
| 6S41-1 | *gtrAB* | Chromosome | bactoprenol-linked glucose translocase/flippase | - |
|  | *rcsB* |  | transcriptional regulator |  |
|  | *ompA* |  | outer membrane protein |  |
|  | *csgBDFG* |  | minor curlin subunit precursor curli nucleator protein/DNA-binding transcriptional regulator/curli production assembly/transport protein |  |
|  | *tssALMJ*, *clpV/tssH*, *hcp2/tssD2* |  | type VI secretion system protein/ ATPase |  |
|  | *gnd* |  | 6-phosphogluconate dehydrogenase |  |
|  | *entBCDEFS* |  | isochorismatase/isochorismate synthase 1/phosphopantetheinyl transferase component of enterobactin synthase multienzyme complex/23-dihydroxybenzoate-AMP ligase component of enterobactin synthase multienzyme complex/enterobactin synthase multienzyme complex component ATP-dependent/enterobactin exporter iron-regulated |  |
|  | *fepABCDG* |  | ferrienterobactin outer membrane transporter /ferrienterobactin ABC transporter periplasmic binding protein |  |
|  | *fes* |  | enterobactin/ferric enterobactin esterase |  |
|  | *acrB* |  | acriflavine resistance protein |  |
|  | *kpsMS* |  | capsule polysaccharide export protein/polysialic acid transport protein |  |
|  | *gspCDEFGHIJKLM* |  | general secretion pathway protein |  |
|  | *aslA* |  | putative arylsulfatase |  |
|  | *cseA* | p1-6S41-1 | adhesin protein | IncFII |
|  | - | p2-6S41-1 | - | IncR/ IncX1 |
|  | - | p6S41-1 | - | IncX4 |
|  | - | p4-6S41-1 | - | no hit found |
|  | - | p5-6S41-1 | - | no hit found |
|  | - | p6-6S41-1 | - | no hit found |
| 5ZF15-2-1 | *ompA* | Chromosome | outer membrane protein | - |
|  | *csgBDFG* |  | minor curlin subunit precursor curli nucleator protein/DNA-binding transcriptional regulator/curli production assembly/transport protein |  |
|  | *astA* |  | heat-stable enterotoxin |  |
|  | *tssAJLM*, *clpV/tssH*, *hcp2*/*tssD2* |  | type VI secretion system protein/ ATPase |  |
|  | *gnd* |  | 6-phosphogluconate dehydrogenase |  |
|  | *entBCDEFS* |  | isochorismatase/isochorismate synthase 1/phosphopantetheinyl transferase component of enterobactin synthase multienzyme complex/23-dihydroxybenzoate-AMP ligase component of enterobactin synthase multienzyme complex/enterobactin synthase multienzyme complex component ATP-dependent/enterobactin exporter iron-regulated |  |
|  | *fepABCDG* |  | ferrienterobactin outer membrane transporter /ferrienterobactin ABC transporter periplasmic binding protein |  |
|  | *fes* |  | enterobactin/ferric enterobactin esterase |  |
|  | *acrB* |  | acriflavine resistance protein |  |
|  | *gspCDEFGHIJKLM* |  | general secretion pathway protein |  |
|  | *kpsCDEFMSU* |  | capsule polysaccharide export protein/polysialic acid transport protein precursor/polysialic acid capsule expression protein/3-deoxy-manno-octulosonate cytidylyltransferase |  |
|  | *aslA* |  | putative arylsulfatase |  |
|  | *gtrA* |  | bactoprenol-linked glucose translocase/flippase |  |
|  | *rcsB* |  | transcriptional regulator |  |
|  | - | p15ZF15-2-1 | - | IncFIB |
|  | - | p5ZF15-2-1 | - | IncI2 |
|  | - | p35ZF15-2-1 | - | IncX1 |
|  | - | p45ZF15-2-1 | - | no hit found |

**Table S2** Antibiotic Resistance Genes in Plasmids of *E. fergusonii* 6S41-1

|  | Drug class | Coding genes | Position |
| --- | --- | --- | --- |
| p2-6S41-1 | Aminoglycoside | *aac(3)-IId* | 36-896 |
|  | Florfenicol | *floR* | 21,492-22,705 |
| p6S41-1 | Colistin | *mcr-1.1* | 13,480-15,105 |
|  | Ciprofloxacin | *qnrS1* | 22,939-23,595 |
| p6-6S41-1 | Tetracycline | *tet(A)* | 1-1,121 |

**Table S3** Amino acid variations of two-component systems and AcrAB-TolC pump system.

| Strain | Two-component systems | | | | | AcrAB-TolC pump system | | | | | |
| --- | --- | --- | --- | --- | --- | --- | --- | --- | --- | --- | --- |
|  | PmrA | PmrB | PhoP | PhoQ | MgrB | TolC | AcrA | AcrB | SoxR | SoxS | MarA |
| 6S41-1 | E144G |  |  | K64R | M1V |  |  |  |  |  |  |
|  |  |  |  | I340V |  |  |  |  |  |  |  |
|  |  |  |  | K376Q |  |  |  |  |  |  |  |
| 5ZF15-2-1 | E144G |  |  | **T299I** | M1V |  |  |  |  |  |  |
|  |  |  |  | I340V |  |  |  |  |  |  |  |
|  |  |  |  | K376Q |  |  |  |  |  |  |  |
|  |  |  |  | G482V |  |  |  |  |  |  |  |

**Note**: Amino acid substitution be predicted to affect protein function is indicated by bold type.

**Table S4** Characteristics of 114 *E. fergusonii* genome sequences analyzed in this study. The data were downloaded from Pathogen Detection (<https://www.ncbi.nlm.nih.gov/pathogens/>) and NCBI database

| BioSample | Assembly accession | Location | Isolation Source | Collection Date |
| --- | --- | --- | --- | --- |
| SAMN16357580 | GCA_019047545.1 | Germany | not collected | not collected |
| SAMN03734431 | GCA_008064915.1 | USA | not collected | not collected |
| SAMN03734432 | GCA_012811895.1 | USA | not collected | not collected |
| SAMN03744015 | GCA_012811775.1 | USA | not collected | not collected |
| SAMN01916802 | GCA_008064895.1 | not collected | Feces | not collected |
| SAMN17899904 | GCA_017890065.1 | USA | Cecal | 2020 |
| SAMN03389646 | GCA_000968895.2 | Canada | Ground beef | 2014 |
| SAMN03744123 | GCA_012811855.1 | USA | not collected | not collected |
| SAMN01916852 | GCA_008064875.1 | USA | Feces | not collected |
| SAMN07159037 | GCA_012466685.1 | USA | not collected | not collected |
| SAMN20033693 | GCA_019219305.1 | China | Feces | 2019 |
| SAMN18140421 | GCA_017890025.1 | USA | Cecal | 2020 |
| SAMN18376170 | GCA_017528495.1 | USA | Cecal | 2020 |
| SAMN18378840 | GCA_017527705.1 | USA | Cecal | 2021 |
| SAMN18378839 | GCA_017527595.1 | USA | Cecal | 2021 |
| SAMN18435363 | GCA_017566905.1 | USA | Cecal | 2020 |
| SAMEA5852020 | GCA_902387905.1 | not collected | Human gut | not collected |
| SAMN15148151 | GCA_013710195.1 | United Kingdom | Feces | 2017 |
| SAMN15148442 | GCA_013798785.1 | United Kingdom | Feces | 2017 |
| SAMN15148437 | GCA_013799825.1 | United Kingdom | Feces | 2017 |
| SAMN15148432 | GCA_013800985.1 | United Kingdom | Feces | 2017 |
| SAMN15148383 | GCA_013812875.1 | United Kingdom | Feces | 2017 |
| SAMN15148384 | GCA_013813425.1 | United Kingdom | Feces | 2017 |
| SAMN15148324 | GCA_013819565.1 | United Kingdom | Feces | 2017 |
| SAMN15148320 | GCA_013820225.1 | United Kingdom | Feces | 2017 |
| SAMN15148317 | GCA_013820465.1 | United Kingdom | Feces | 2017 |
| SAMN15148308 | GCA_013821535.1 | United Kingdom | Feces | 2017 |
| SAMN15148304 | GCA_013822105.1 | United Kingdom | Feces | 2017 |
| SAMN15148247 | GCA_013834165.1 | United Kingdom | Feces | 2017 |
| SAMN15148244 | GCA_013834525.1 | United Kingdom | Feces | 2017 |
| SAMN15148237 | GCA_013836325.1 | United Kingdom | Feces | 2017 |
| SAMN15148225 | GCA_013838265.1 | United Kingdom | Feces | 2017 |
| SAMN15148224 | GCA_013838465.1 | United Kingdom | Feces | 2017 |
| SAMN15148209 | GCA_013886195.1 | United Kingdom | Feces | 2017 |
| SAMN15148208 | GCA_013886335.1 | United Kingdom | Feces | 2017 |
| SAMN15148162 | GCA_013892435.1 | United Kingdom | Feces | 2017 |
| SAMN15148152 | GCA_013893835.1 | United Kingdom | Feces | 2017 |
| SAMN15148146 | GCA_013894595.1 | United Kingdom | Feces | 2017 |
| SAMN15148066 | GCA_013899685.1 | United Kingdom | Feces | 2017 |
| SAMN15148055 | GCA_013899845.1 | United Kingdom | Feces | 2017 |
| SAMN05464554 | GCA_002965025.1 | USA | Feces | 1987 |
| SAMN05464487 | GCA_002965935.1 | USA | Pleural cavity | 1983 |
| SAMN02596695 | GCA_000190495.1 | not collected | not collected | not collected |
| SAMN03400068 | GCA_000968885.2 | Canada | Chopped kale | 2014 |
| SAMN16304066 | GCA_015676145.1 | Canada | Feces | 2017 |
| SAMN15148326 | GCA_013819085.1 | United Kingdom | Feces | 2017 |
| SAMN15148325 | GCA_013819325.1 | United Kingdom | Feces | 2017 |
| SAMN15148313 | GCA_013820845.1 | United Kingdom | Feces | 2017 |
| SAMN15148153 | GCA_013928365.1 | United Kingdom | Feces | 2017 |
| SAMN15148376 | GCA_014042125.1 | United Kingdom | Feces | 2017 |
| SAMN15148372 | GCA_014042145.1 | United Kingdom | Feces | 2017 |
| SAMN15148375 | GCA_014104595.1 | United Kingdom | Feces | 2017 |
| SAMN15148373 | GCA_014104615.1 | United Kingdom | Feces | 2017 |
| SAMN15148374 | GCA_014104625.1 | United Kingdom | Feces | 2017 |
| SAMN15148315 | GCA_014104845.1 | United Kingdom | Feces | 2017 |
| SAMN15148245 | GCA_014105005.1 | United Kingdom | Feces | 2017 |
| SAMN15148235 | GCA_014104995.1 | United Kingdom | Feces | 2017 |
| SAMN15148032 | GCA_014105935.1 | United Kingdom | Feces | 2017 |
| SAMN03744014 | GCA_012811875.1 | USA | not collected | not collected |
| SAMN16357201 | GCA_016128235.1 | USA | not collected | not collected |
| SAMN18140327 | GCA_017890045.1 | USA | Cecal | 2020 |
| SAMN18378762 | GCA_017527765.1 | USA | Cecal | 2021 |
| SAMEA3268854 | GCA_012779555.1 | Denmark | not collected | 2011 |
| SAMEA3138228 | GCA_000026225.1 | not collected | not collected | not collected |
| SAMN18140547 | GCA_017889985.1 | USA | Cecal | 2020 |
| SAMN18140549 | GCA_017889965.1 | USA | Cecal | 2020 |
| SAMN18987026 | GCA_018259015.1 | United Kingdom | Human | 2021 |
| SAMEA3268857 | GCA_012779675.1 | Denmark | not collected | 2011 |
| SAMN15148429 | GCA_013801425.1 | United Kingdom | Feces | 2017 |
| SAMN15148427 | GCA_013801845.1 | United Kingdom | Feces | 2017 |
| SAMN15148386 | GCA_013813205.1 | United Kingdom | Feces | 2017 |
| SAMN20152536 | GCA_019198775.1 | USA | Cecal | 2021 |
| SAMN18375737 | GCA_018018155.1 | USA | Cecal | 2020 |
| SAMN18375734 | GCA_018018135.1 | USA | Cecal | 2020 |
| SAMN18377570 | GCA_017528395.1 | USA | Cecal | 2021 |
| SAMN18378761 | GCA_017527845.1 | USA | Cecal | 2021 |
| SAMN02470542 | GCA_000191665.1 | Canada | Broiler chicken | 2004 |
| SAMN03400083 | GCA_000968905.2 | Canada | Beef trim | 2014 |
| SAMEA5771506 | GCA_902167795.1 | not collected | not collected | not collected |
| SAMN11392254 | GCA_015330985.1 | United Kingdom | Human | 2019 |
| SAMN14604091 | GCA_013171325.1 | China | Feces | 2019 |
| SAMEA6968676 | GCA_903932165.1 | Australia | Clade | 2001 |
| SAMN15148431 | GCA_013591455.1 | United Kingdom | Feces | 2017 |
| SAMN18376173 | GCA_017528565.1 | USA | Cecal | 2020 |
| SAMN18377582 | GCA_017528445.1 | USA | Cecal | 2020 |
| SAMN18377577 | GCA_017528365.1 | USA | Cecal | 2020 |
| SAMN18435484 | GCA_018017875.1 | USA | Cecal | 2020 |
| SAMN10145472 | GCA_003944555.1 | China | Feces | 2017 |
| SAMN14655852 | GCA_012909925.1 | United Kingdom | Human | 2020 |
| SAMN13824823 | GCA_009903715.2 | Brazil | Cloacal swab | 2019 |
| SAMN14891541 | GCA_013106575.1 | United Kingdom | Human | 2020 |
| SAMEA6967831 | GCA_903932095.1 | France | Feces | 2004 |
| SAMN15147969 | GCA_013714535.1 | United Kingdom | Feces | 2017 |
| SAMN15147968 | GCA_013714825.1 | United Kingdom | Feces | 2017 |
| SAMN15147967 | GCA_013715025.1 | United Kingdom | Feces | 2017 |
| SAMN15147971 | GCA_013923185.1 | United Kingdom | Feces | 2017 |
| SAMN08534259 | GCA_002951525.1 | China | not collected | 2016 |
| SAMN20394500 | GCA_019338245.1 | USA | Cecal | 2021 |
| SAMN18376179 | GCA_017528585.1 | USA | Cecal | 2020 |
| SAMN18378057 | GCA_017528185.1 | USA | Cecal | 2021 |
| SAMN07682644 | GCA_013038665.1 | China | Sludge | 2016 |
| SAMN15147982 | GCA_013713585.1 | United Kingdom | Feces | 2017 |
| SAMN15147978 | GCA_014106295.1 | United Kingdom | Feces | 2017 |
| SAMN07682645 | GCA_013038725.1 | China | Anus swab | 2016 |
| SAMN07682646 | GCA_013038705.1 | China | Anus swab | 2016 |
| SAMN15148269 | GCA_013596835.1 | United Kingdom | Feces | 2017 |
| SAMN15148292 | GCA_013825725.1 | United Kingdom | Feces | 2017 |
| SAMN15148274 | GCA_013829265.1 | United Kingdom | Feces | 2017 |
| SAMN15148291 | GCA_014104895.1 | United Kingdom | Feces | 2017 |
| SAMN15148286 | GCA_014104915.1 | United Kingdom | Feces | 2017 |
| SAMN08534257 | GCA_002951495.1 | China | not collected | 2016 |
| SAMN10531910 | GCA_003944565.2 | China | Feces | 2017 |
| SAMEA2612497 | GCA_900450565.1 | not collected | not collected | 1800/2017 |
| SAMN14688229 | GCA_012972665.1 | Bangladesh | not collected | 2016 |

**Figure S1.** Correlation matrix of average nucleotide identity for the 116 *E. fergusonii* genomes obtained by pyani.


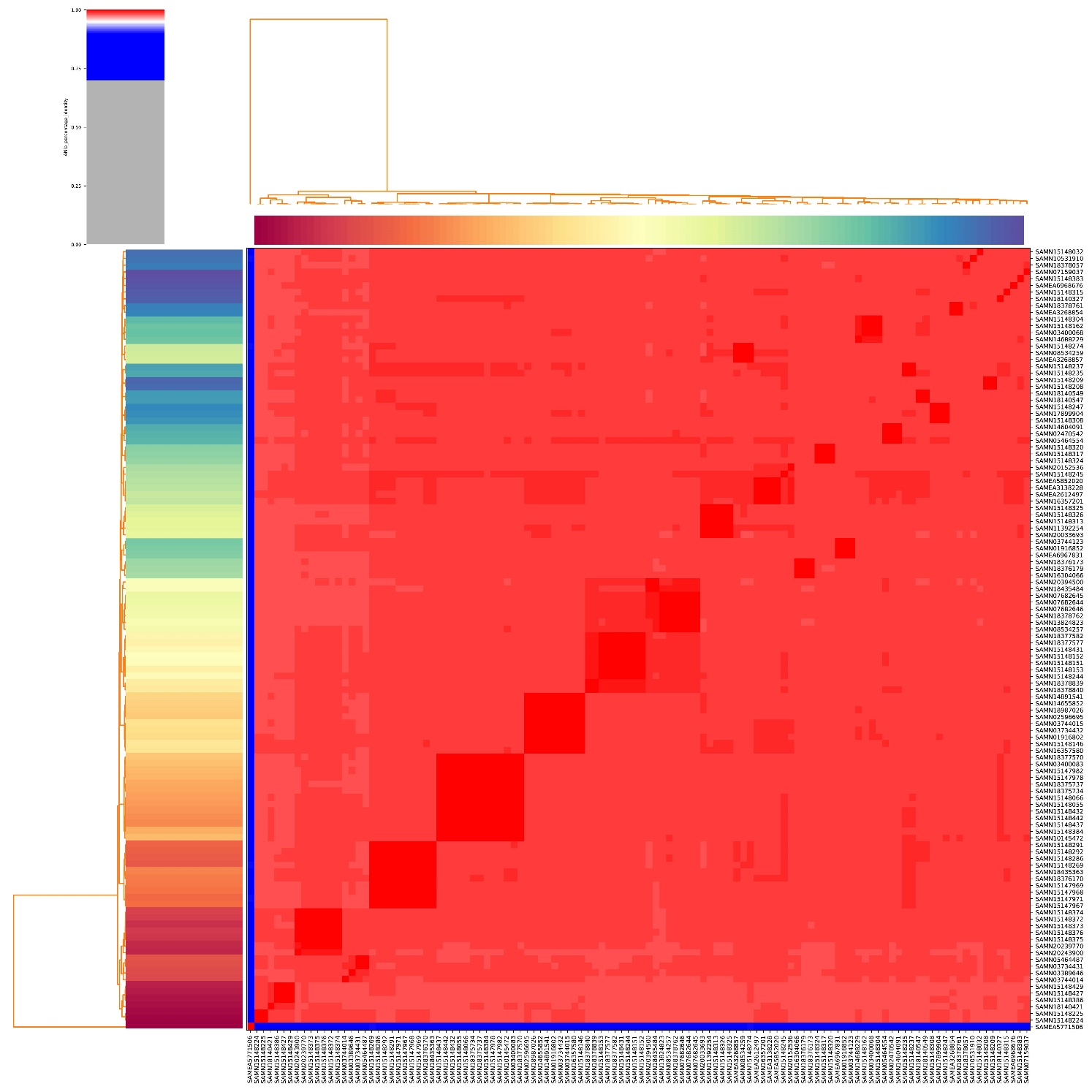

Supplement: Supplementary file 1 [file DataSheet_1.docx]
